# HADHA promotes ovarian cancer outgrowth via up-regulating CDK1

**DOI:** 10.1186/s12935-023-03120-4

**Published:** 2023-11-20

**Authors:** Yinglan Liu, Ying Xiong

**Affiliations:** 1 Department of Obsdetrics and Gynecology, First Affiliated Hospital of Harbin Medical University, No.23, Youzheng Road, Harbin city, 150001 Heilongjiang Province China; 2 Department of Gynecology, State Key Laboratory of Oncology in South China, Guangdong Provincial Clinical Research Center for Cancer, Sun Yat-Sen University Cancer Center, No.651, Dongfengdong Road, Guangzhou, 5100160 Guangdong Province China

**Keywords:** Ovarian cancer, HADHA, CDK1, Ubiquitination

## Abstract

**Background:**

Ovarian cancer, a prevalent cause of cancer-related mortality among gynecological cancers, still lacks a clear understanding of its pathogenesis. In this study, our objective was to investigate the functional roles and pathogenic mechanisms of HADHA in ovarian cancer.

**Methods:**

We utilized an ovarian cancer tissue microarray and three ovarian cancer cell lines (HO-8910, A2780, and SK-OV-3) for our analysis. Lentiviral-mediated short hairpin RNA (shRNA) was employed to interfere with HADHA expression in ovarian cancer cells. Various cellular events associated with tumor development were assessed using techniques such as Celigo cell counting assay, wound healing assay, Transwell assay, and flow cytometry analysis. Additionally, xenograft tumor models were developed to visualize the impacts of HADHA/CDK1 on ovarian cancer progression.

**Results:**

Our data revealed significant HADHA overexpression in both ovarian cancer tissues and cell lines. Patients with elevated HADHA levels tended to experience poor survival outcomes. Moreover, HADHA upregulation correlated with several pathological parameters, including pathological stage, tumor size, tumor infiltrate, metastasis, and recurrence. Loss-of-function experiments targeting HADHA demonstrated that its suppression in ovarian cancer cells hindered cell growth and migration, while promoting apoptosis. To elucidate the underlying mechanism by which HADHA regulates ovarian cancer, we identified CDK1 as a target of HADHA. HADHA upregulated CDK1 expression by inhibiting its ubiquitination-dependent proteasomal degradation. Significantly, the overexpression of CDK1 reversed the impaired cell development caused by HADHA depletion, both in vitro and in vivo.

**Conclusion:**

Our study highlights the involvement of HADHA in ovarian cancer tumorigenesis and suggests its potential as a promising prognostic marker in ovarian cancer. Through its regulation of CDK1, HADHA influences critical cellular processes in ovarian cancer, providing insights into its pathogenic mechanism.

**Supplementary Information:**

The online version contains supplementary material available at 10.1186/s12935-023-03120-4.

## Background

Globally, ovarian cancer accounts for approximately 239,000 new cases and 152,000 deaths annually, making it the leading cause of death among gynecological cancers in most developed countries [1]. The World Health Organization (WHO) classifies epithelial ovarian carcinomas (EOC) into subtypes such as serous carcinomas (SC), mucinous carcinomas (MC), endometrioid carcinoma (EC), clear cell carcinoma (CCC), transitional cell Brenner tumors, mixed types, and undifferentiated types [2]. Despite their differences in etiology, morphology, and molecular biology, these subtypes are generally treated as a single disease entity. The current standard treatment for ovarian cancer involves cytoreductive surgery followed by a combination of platinum-based and taxane-based chemotherapy [3, 4]. The initial response rate to this first-line therapy is around 80–90%; however, a majority of patients eventually experience relapse and develop resistance to chemotherapy, resulting in a less than 35% 5-year survival rate [4]. Therefore, there is an urgent need to identify additional molecular targets for therapy to improve overall prognosis in ovarian cancer. Furthermore, further research is necessary to identify potential prognostic markers that can monitor recurrence or progression in localized ovarian cancer patients.

The alpha subunit of the mitochondrial trifunctional protein (TFP), known as Hydroxyacyl-CoA dehydrogenase alpha subunit (HADHA), plays a crucial role in the final steps of mitochondrial β-oxidation of long-chain fatty acids. The complete mitochondrial TFP is composed of four alpha subunits (HADHA) and four beta subunits (HADHB). HADHA is responsible for catalyzing the activities of long-chain 3-hydroxyacylcoenzyme A dehydrogenase (LCHAD) and enoylcoenzyme A hydratase (ECH), while HADHB carries out the 3-ketoacyl-coenzyme A thiolase (KACT) activity [5]. Mutations in HADHA or a reduction in its enzyme activity to below 50% of normal have been associated with severe manifestations of LCHAD deficiency, such as hypoglycemia, cardiomyopathy, and sudden death [6]. However, recent research has revealed that HADHA, as a key lipid metabolic enzyme, also plays a novel role in the development of various cancers, including lung cancer [7], renal cell carcinoma [8], hepatocellular carcinoma [9], malignant lymphoma [10] and breast cancer [11]. Despite these findings in other cancer types, our knowledge of the specific roles and potential mechanisms of HADHA in ovarian cancer is currently limited. Further research is needed to explore the involvement of HADHA in ovarian cancer and elucidate its precise mechanisms of action in this context.

In this study, we conducted an analysis of HADHA expression levels in ovarian cancer to elucidate its potential functions. Additionally, we assessed the prognostic value of HADHA in ovarian cancer and investigated the underlying downstream mechanism of its action. Our findings establish HADHA as a significant tumor-promoting factor in the development of ovarian cancer.

## Methods

### Ethical approval

The study received approval from the Department of Obstetrics and Gynecology of the First Affiliated Hospital of Harbin Medical University (Approval No. 2,021,070). All procedures conducted in this study adhered to the ethical guidelines outlined in the Declaration of Helsinki.

### Tissues, cell lines and cell culture

In this study, we utilized an ovarian cancer tissue microarray (TMA) consisting of 105 tumor tissues and 19 normal tissues. To investigate cellular responses and mechanisms, we employed human normal ovarian epithelial cells IOSE80 and three ovarian cancer cell lines (HO-8910, A2780, and SK-OV-3), which were obtained from the American Type Culture Collection (ATCC). These cells were cultured in PRIM-1640 medium (Hyclone, UT, USA) supplemented with 10% fetal bovine serum (FBS) (GIBCO BRL, BRA, USA), and 1 × 10^5^ IU/L penicillin and streptomycin (Beyotime Biotechnology, SH, China). The cell culture was maintained under conditions of 5% CO_2_ and 95% humidity.

### Immunohistochemistry (IHC) analysis and evaluation

IHC analysis was performed as described previously [12]. Immunostained slides were scanned and analyzed using a fluorescent microscope (Olympus, Japan). To ensure accuracy, two pathologists, who were blinded to the clinicopathological parameters, independently evaluated the immunostaining of the TMA slides. The immunostaining was evaluated by recording the percentage of cells showing positive staining. Subsequently, the mean percentage of cells with positive staining was calculated. Based on the median percentage of positive cells for each antibody, the protein expression results were classified into two groups: the low group (no staining or less than the median value) and the high group (equal to or greater than the median value). More detailed information about the antibodies used can be found in Table S1.

### Transfection

To achieve HADHA knockdown in ovarian cancer cells, we employed lentiviral-mediated short hairpin RNA (shRNA) interference. The shRNA was designed to specifically target HADHA and was delivered via lentiviral vectors. For CDK1 overexpression, we utilized CDK1 as a template and designed corresponding primers for amplification. The amplified CDK1 product was then used for the construction of an overexpression plasmid.

### Real-time quantitative polymerase chain reaction (RT-qPCR)

The total RNA from IOSE80, HO-8910, A2780, and SK-OV-3 cells was extracted using TRIzol reagent (Sigma). The quality of the extracted RNA was assessed using a Nanodrop 2000 spectrophotometer (Thermo Fisher Scientific). Quantitative real-time PCR (qPCR) was performed with the SYBR Green mastermix Kit (Vazyme), and the 2^−ΔΔCt^ method was employed for relative quantitative analysis of gene expression data. GAPDH was used as an internal control, and the specific primer sequences used were detailed in Table S2.

### Western blot assay and co-immunoprecipitation (Co-IP)

For the protein analysis, HO-8910 and SK-OV-3 cells were lysed in ice-cold RIPA buffer (Millipore). The total protein concentration in the lysates was determined using the BCA Protein Assay Kit (#23,225, HyClone-Pierce). Subsequently, 20 µg of protein from each sample was separated by 10% SDS-PAGE (Invitrogen) and transferred onto PVDF membranes. Primary antibodies and secondary antibodies were separately incubated with the membranes at 4 °C. Protein blots were visualized using enhanced Immobilon Western Chemiluminescent HRP Substrate (Millipore), and signal densities were quantified using ImageJ software (National Institute of Health).

Additionally, for the co-immunoprecipitation (co-IP) assay, proteins from HO-8910 cells were collected and immunoprecipitated using either anti-IgG or anti-HADHA antibodies. Subsequently, the immunoprecipitated proteins were subjected to western blot analysis using antibodies against HADHA and CDK1. The anti-IgG antibody was used as the control group for immunoprecipitation. Detailed information about the antibodies used in these experiments can be found in Table S1.

### Celigo cell counting

After the transfection process, HO-8910 and SK-OV-3 cells were harvested and plated into 96-well plates at a cell density of 1,000 cells per well. These cells were then cultured in their respective growth media containing 10% FBS at a temperature of 37 °C in an atmosphere with 5% CO_2_. Cell counting was carried out using a Celigo image cytometer, which is a specialized instrument manufactured by Nexcelom Bioscience. This counting procedure was conducted over a span of 5 days.

### Wound healing assay

HO-8910 and SK-OV-3 cells transfected with lentivirus were plated in 96-well dishes at a density of 3 × 10^4^ cells per well and allowed to grow until they reached a confluent state. Subsequently, scratches were created across the cell layers, and any floating cells were carefully washed away. Photographs of the scratched areas were captured using a fluorescence microscope, and the cell migration rate for each experimental group was calculated.

### Transwell assay

The Transwell assay was conducted using Corning Transwell inserts (Corning). In this assay, approximately 5 × 10^4^ exponentially growing HO-8910 and SK-OV-3 cells were placed in the upper chamber, while 600 µL of complete medium was added to the lower chamber. The cells were then allowed to incubate for 24 h at 37 °C in an environment with 5% CO_2_. Following the incubation period, non-metastatic cells were carefully removed from the upper chamber using a cotton swab, leaving behind the metastatic cells. Subsequently, the metastatic cells were fixed and stained. The migration ability of the cells was then assessed and analyzed.

### Flow cytometry assay

Lentivirus transfected HO-8910 and SK-OV-3 were inoculated into 6-well plates with 2 mL of culture medium per well in triplicate. The cells were further cultured for a period of 5 days. After the incubation period, the cells were collected, trypsinized to detach them from the plate, and washed with ice-cold D-Hanks solution at a temperature of 4 °C. Subsequently, the cells were pelleted by centrifugation at 1,000 × g. The cell pellet was then resuspended in binding buffer, and 10 µL of Annexin V-APC (eBioscience) was added for staining. This staining process was carried out in the dark to prevent light interference. Apoptosis analysis was performed using a FACSCalibur flow cytometer (BD Biosciences).

### Identified peptide mass spectrometry (IPMS)

HO-8910 cells were first washed with phosphate-buffered saline (PBS). Subsequently, the cells were lysed using immunoprecipitation lysis buffer that contained 100 µM phenylmethylsulfonyl fluoride (PMSF). The resulting cell lysates were then used for immunoprecipitation. This process involved the use of 50 µl of protein A-agarose, along with either anti-HADHA antibody (dilution: 1:50) or normal rabbit IgG (concentration: 1 µg/ml). The proteins that were found to interact with HADHA, the target protein, were identified using mass spectrometry. This identification procedure was carried out using equipment and techniques provided by Applied Protein Technology.

### Bioinformatics analysis

To analyze the differential expression of HADHA and CDK1 in ovarian cancer vs. normal samples, we utilized several datasets from the Gene Expression Omnibus (GEO) database: GSE66957 (https://www.ncbi.nlm.nih.gov/geo/query/acc.cgi?acc=GSE66957), GSE40595 (https://www.ncbi.nlm.nih.gov/geo/query/acc.cgi?acc=GSE40595), GSE36668 (https://www.ncbi.nlm.nih.gov/geo/query/acc.cgi?acc=GSE36668), GSE69428 (https://www.ncbi.nlm.nih.gov/geo/query/acc.cgi?acc=GSE69428), GSE54388 (https://www.ncbi.nlm.nih.gov/geo/query/acc.cgi?acc=GSE54388), GSE105437 (https://www.ncbi.nlm.nih.gov/geo/query/acc.cgi?acc=GSE105437), and GSE26712 (https://www.ncbi.nlm.nih.gov/geo/query/acc.cgi?acc=GSE26712). We preprocessed the raw data using the R package ‘affy’ to obtain RMA-normalized matrices and performed differential expression analysis between ovarian cancer and normal samples using the R package ‘limma’. Using the Spearman method, we calculated the expression correlation of HADHA and CDK1 in ovarian cancer samples across various datasets.

### Mouse model assay

The mice experiment was conducted in accordance with approved ethical protocols. In this experiment, either 4 × 10^6^ transfected HO-8910 cells or control cells were subcutaneously injected into specific pathogen-free BALB/c nude mice. A total of 20 mice were used, with 10 mice randomly assigned to each experimental group. The mice were sourced from Beijing Vital River Laboratory Animal Technology Co., Ltd. Throughout the study, the size and weight of the tumors were regularly monitored twice a week. Tumor volume was calculated using the formula V = π/6 × L × W^2^, where “L” represents the longest dimension of the tumor and “W” denotes the dimension perpendicular to its length. After 22 days post-injection, all mice were anesthetized using 0.7% sodium pentobarbital (administered at a rate of 10 µL per gram of body weight) from SIGMA. Following anesthesia, the mice were humanely euthanized. Tumor tissues were then surgically removed and collected for further analysis. Thin sections of approximately 5 mm in thickness were prepared from the collected tumor tissues and subjected to staining for Ki-67.

### Statistical analysis

All cell experiments, data were obtained from triplicate measurements, and the data are presented as the mean ± standard deviation (SD). Statistical significance was assessed using various methods, including Student’s t-Test or ANOVA test for comparison among multiple groups, Rank Sum test analysis, Mann-Whitney U analysis for non-parametric data, Spearman Rank correlation analysis to evaluate relationships between variables, and the Kaplan-Meier method for survival analysis. The statistical analysis was performed using statistical software packages, specifically SPSS 19.0 by IBM and GraphPad Prism 7.0 by Graphpad Software. A significance threshold of *P* < 0.05 was considered to determine statistical significance in the results.

## Results

### HADHA was frequently upregulated in ovarian cancer

**Fig. 1:**
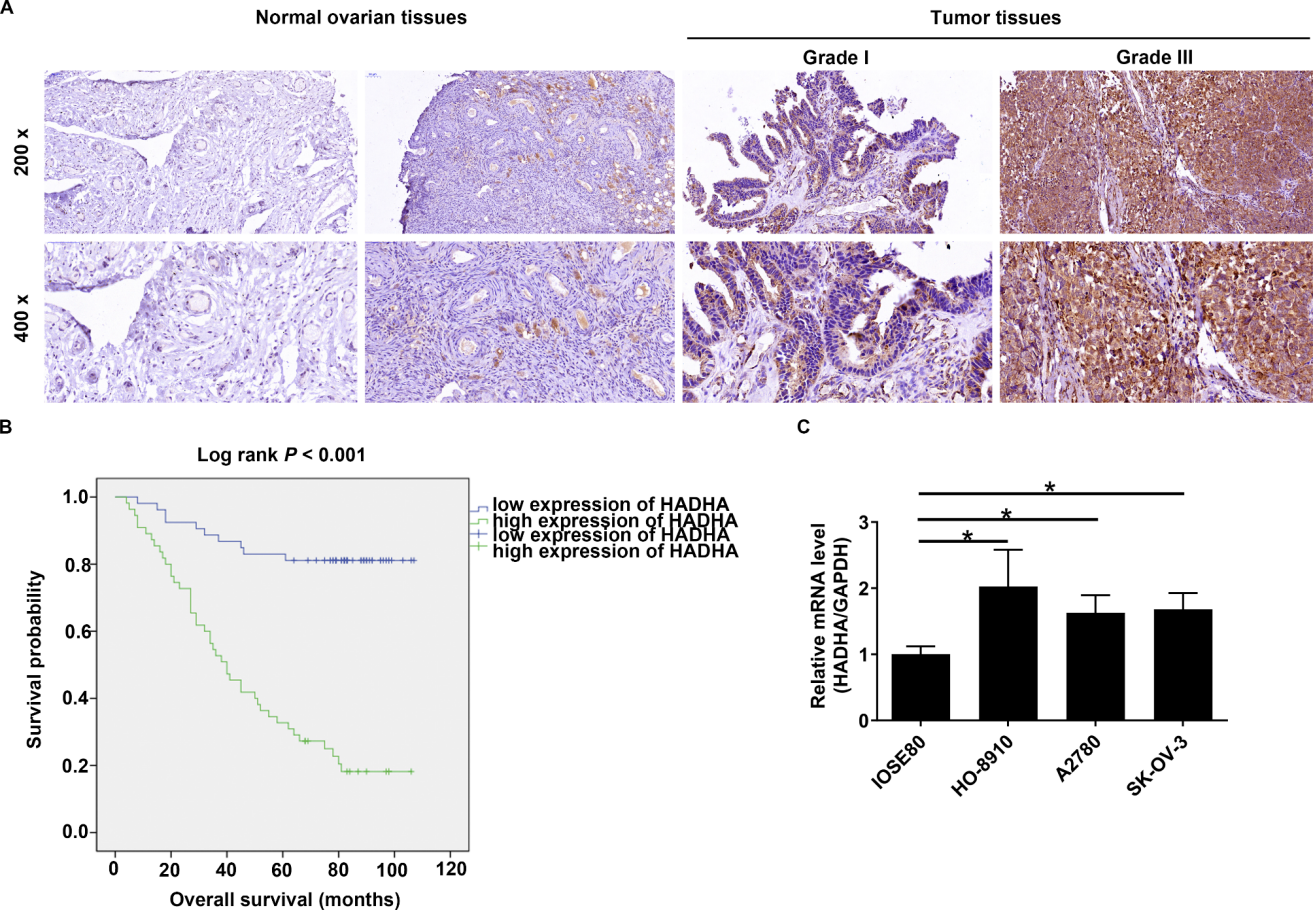
HADHA was frequently upregulated in ovarian cancer. **(A)** IHC analysis stained HADHA density in ovarian cancer and normal tissues from a TMA. **(B)** The prognostic significance of HADHA in ovarian cancer. **(C)** HADHA mRNA level in three ovarian cancer cell lines HO-8910, A2780, SK-OV-3 cells, as well as normal ovarian epithelial cells IOSE80. * *P* < 0.05

In order to elucidate the roles of HADHA in ovarian cancer pathogenesis, our initial objective was to assess its protein expression in ovarian cancer specimens and compare it with healthy controls. Immunohistochemical (IHC) staining of an ovarian cancer tissue microarray revealed that HADHA expression was elevated in 50.9% of tumor samples (N = 108, Fig. 1A and Table 1). In contrast, in normal ovarian tissues, HADHA expression was markedly reduced, with a 100% reduction observed in 19 healthy control samples (Fig. 1A and Table 1). Additionally, we conducted Mann-Whitney U analysis, which demonstrated a statistically significant positive correlation between HADHA levels and various clinicopathological parameters, including pathological stage, tumor size, tumor infiltrate, metastasis and recurrence of state (Table 2). Notably, the upregulation of HADHA was associated with decreased survival compared to patients exhibiting low expression of HADHA, as illustrated in Fig. 1B. Simultaneously, we detected elevated HADHA mRNA level in three ovarian cancer cell lines HO-8910, A2780 and SK-OV-3 cells, in comparison to normal ovarian epithelial cells IOSE80. Particularly, HO-8910 and SK-OV-3 cell lines displayed significantly elevated HADHA mRNA levels (Fig. 1C). These data demonstrated that HADHA is frequently upregulated in ovarian cancer, highlighting its potential significance in the disease’s pathogenesis.

**Table 1:** Expression patterns of HADHA in ovarian cancer tissues and normal ovarian tissues revealed in immunohistochemistry analysis

HADHA expression	Tumor tissue		Normal ovarian tissue		*P* value
	Cases	Percentage	Cases	Percentage	
Low	51	48.6%	19	100%	< 0.001
High	54	51.4%	0		

**Table 2:** Relationship between HADHA expression and tumor characteristics in patients with ovarian cancer

Features	No. of patients	HADHA expression		*P* value
		low	high	
All patients	105	51	54	
Age (years)				0.498
≤ 51	52	27	25	
>51	53	24	29	
stage				0.001
1	4	4	0	
2	26	21	5	
3	54	22	32	
4	21	4	17	
Tumor size (cm)				0.026
≤ 12.5	52	31	21	
> 12.5	53	20	33	
Tumor infiltrate				< 0.001
T1	4	4	0	
T2	26	21	5	
T3	75	26	49	
Lymphatic metastasis (N)				< 0.001
N0	76	48	28	
N1	29	3	26	
Metastasis				0.003
M0	84	47	37	
M1	21	4	17	
Recurrence of state				0.001
No	18	15	3	
Yes	87	36	51	

### Depletion of HADHA results in impaired cell viability and migration in vitro

**Fig. 2:**
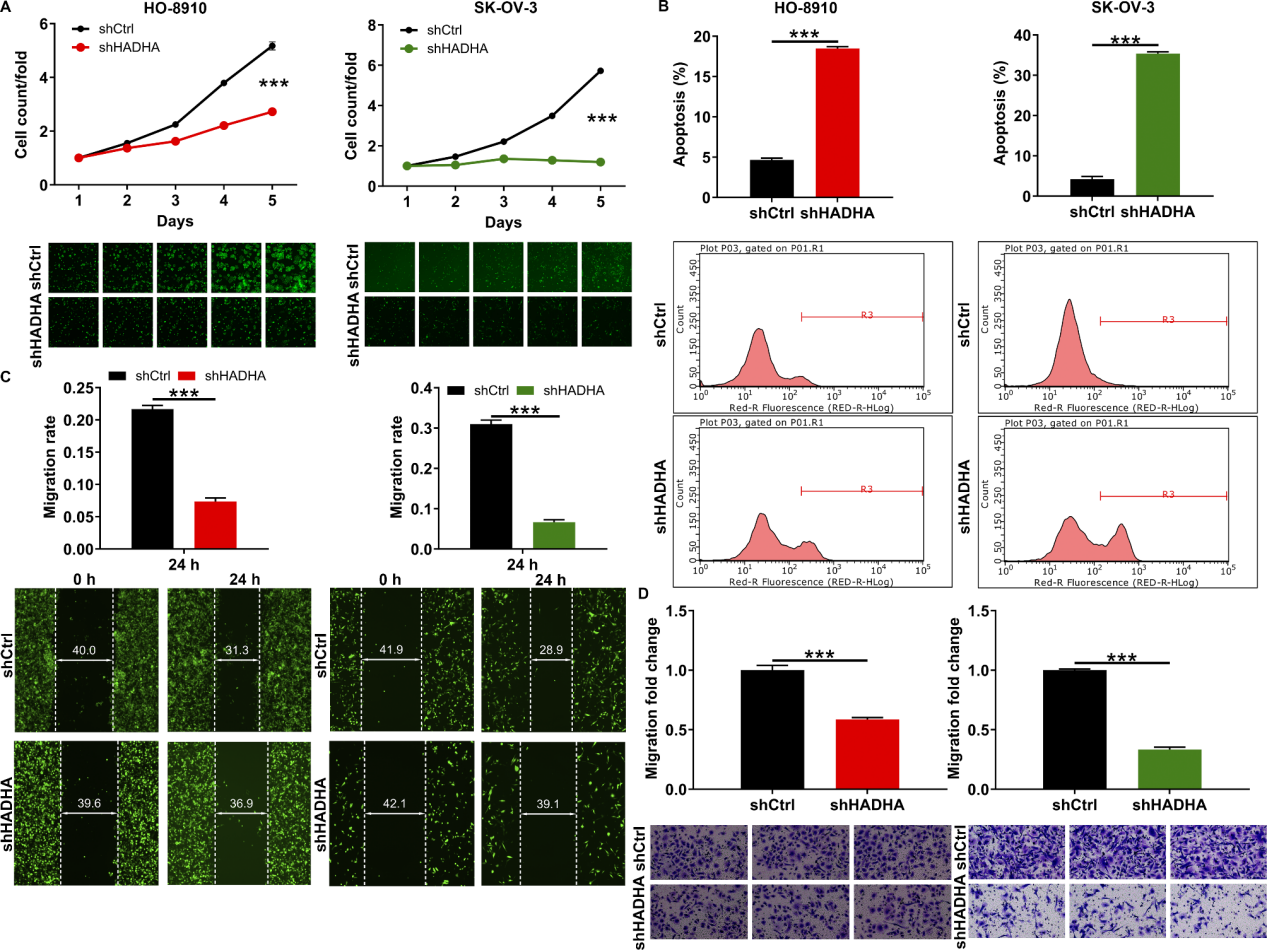
Depletion of HADHA results in impaired cell viability and migration *in vitro.***(A)** Celigo cell counting assay was performed to assess the abilities of cell growth in. **(B)** Flow cytometry experiment visualized the status of cell apoptosis after knocking down HADHA in HO-8910 and SK-OV-3 cells. **(C, D)** Cell migration was evaluated after knocking down HADHA in HO-8910 and SK-OV-3 cells through wound-healing assay **(C)** and transwell assay **(D)**. *** *P* < 0.001

To investigate the consequences of loss of HADHA expression, we established ovarian cancer cell line (HO-8910 and SK-OV-3) with HADHA knockdown using short hairpin RNA (shRNA) that expressed HADHA. The efficacy of the shRNA-mediated knockdown was confirmed through qRT-PCR and western blot assays, as depicted in Figure S1. These assays demonstrated successful and efficient transfection of shHADHA and a negative control lentivirus (shCtrl) into HO8910 and SK-OV-3 cells. Subsequently, we examined the phenotypic changes in these cell models following HADHA knockdown. Utilizing Celigo cell counting assays, we observed that HADHA-deficient cells exhibited a significantly reduced proliferation rate (Fig. 2A). Moreover, HADHA-deficient cells displayed an enhanced apoptotic potential, as evidenced in Fig. 2B. Besides, these cells demonstrated reduced motility in wound-healing assays, with more than a three-fold reduction in mobility observed (Fig. 2C). Similar results were obtained in transwell assay (Fig. 2D). These data demonstrated that depleting HADHA leads to impaired cell viability and migration, while simultaneously promoting apoptosis in vitro. These findings highlight the important role of HADHA in the regulation of key cellular processes associated with ovarian cancer pathogenesis.

### CDK1 is a downstream target of HADHA

**Fig. 3:**
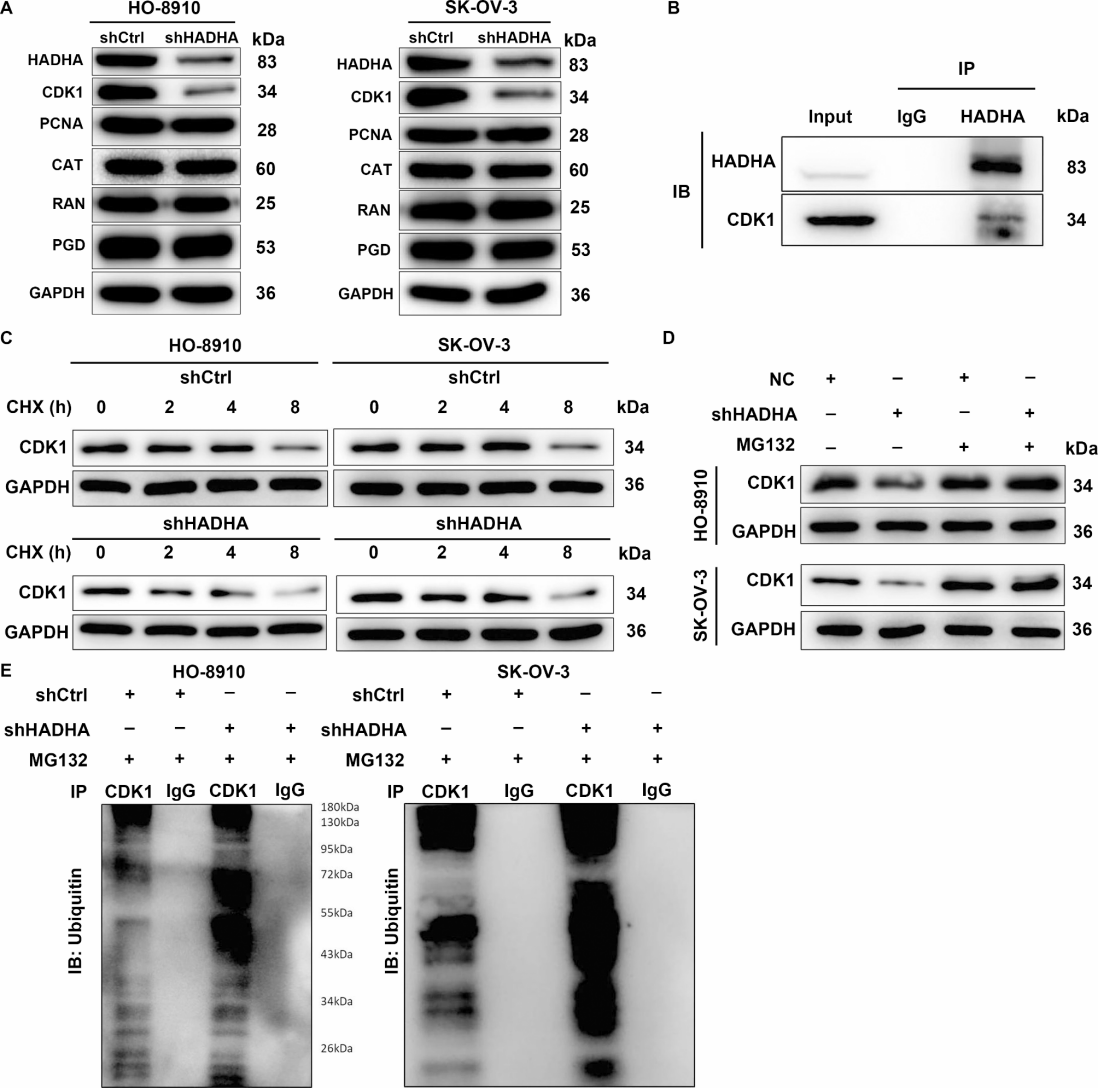
CDK1 is a downstream target of HADHA. **(A)** The levels of HADHA, CDK1, PCNA, CAT, RAN and PGD were detected in HADHA-depleted HO-8910 and SK-OV-3 cells. **(B)** The endogenous relationship between HADHA and CDK1. **(C)** The half-life of CDK1 was analyzed in HO-8910 and SK-OV-3 cells with/without HADHA depletion. **(D)** The expression of CDK1 was detected in HADHA-depleted HO-8910 and SK-OV-3 cells after MG132 treatment. **(D)** The ubiquitination level of CDK1 was detected in immunoprecipitates of CDK1 collected from HADHA-depleted HO-8910 and SK-OV-3 cells

To unravel the mechanisms underlying the impacts of HADHA on ovarian cancer development, we conducted an Identified Peptide Mass Spectrometry (IPMS) analysis to identify proteins that interact with HADHA. Next, we examined the status of these proteins in ovarian cancer and selected the top 5 proteins for further investigation. We assessed the expression levels of these proteins in HADHA knockdown cells, and the results revealed a significant reduction in CDK1 expression, as depicted in Fig. 3A. We also conducted an assessment of the expression levels of HADHA and CDK1 using multicenter high-throughput datasets from the GEO database. The results were presented in Figure S2, which revealed that HADHA exhibited significant upregulation in ovarian cancer samples compared to normal samples within the GSE66957 dataset. Conversely, CDK1 displayed pronounced upregulation in ovarian cancer samples relative to normal samples across multiple datasets, including GSE40595, GSE36668, GSE69428, GSE54388, and GSE26712. Through a comprehensive analysis of these multiple datasets, we identified a positive correlation between the expression levels of HADHA and CDK1 in ovarian cancer samples specifically within the GSE26712 dataset (*P* < 0.05).These led us to hypothesize that CDK1 might be a downstream target of HADHA involved in the regulation of ovarian cancer. To delve deeper into the mechanistic aspects of how the loss of HADHA impacts CDK1 expression, we made an incidental discovery that endogenously expressed HADHA specifically interacts with CDK1, indicating a protein-protein interaction between HADHA and CDK1 (Fig. 3B). To further study the mechanism by which HADHA depletion mediates the reduction in CDK1 protein expression, we treated HADHA-depleted HO8910 and SK-OV-3 cells with cycloheximide (CHX) to assess the half-life of CDK1. Western blotting analysis showed that the inhibition of HADHA accelerated the degradation of CDK1 protein (Fig. 3C). This effect was partially rescued by the addition of the proteasome inhibitor MG132 (Fig. 3D). These findings imply that HADHA may regulate CDK1 through the ubiquitin-proteasome system (UPS). Given that protein ubiquitination is commonly associated with proteasome-mediated degradation [13, 14], we further evaluated the ubiquitination levels of CDK1. As anticipated, HADHA depletion augmented the ubiquitination of CDK1 (Fig. 3E). Thus, we proposed that HADHA could stabilize CDK1 by affecting CDK1 ubiquitination.

### HADHA promoted the progression of ovarian cancer by upregulating CDK1

**Fig. 4:**
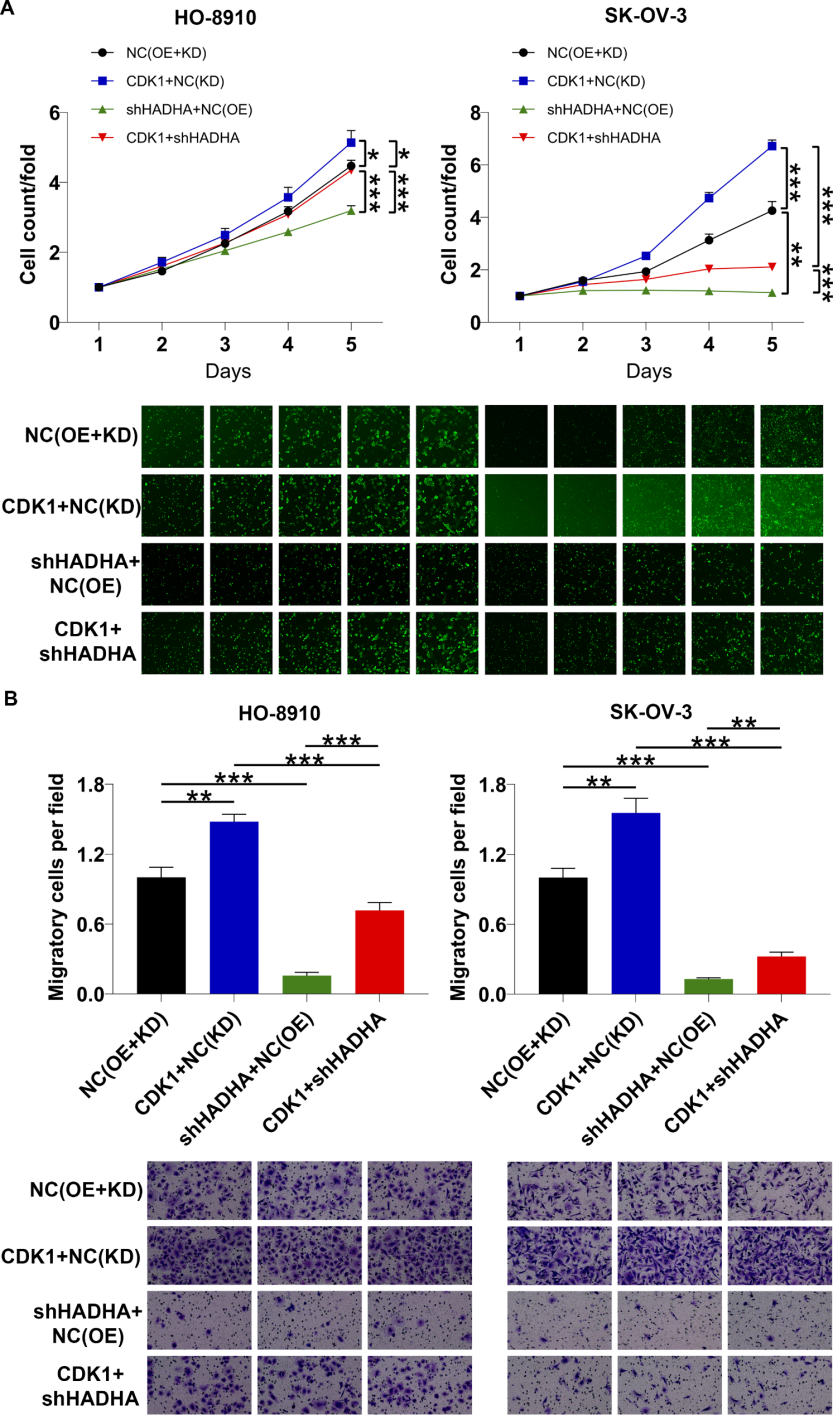
HADHA regulated ovarian cancer cell behaviors by upregulating CDK1. **(A, B)** Celigo cell counting assay and transwell experiment were employed to evaluate the roles of HADHA/CDK1 in ovarian cancer cell proliferation **(A)** and migration **(B)**. NC (OE + KD): Control; CDK1 + NC (KD): CDK1 overexpression; shHADHA + NC (OE): HADHA downregulation; CDK1 + shHADHA: CDK1 overexpression and HADHA downregulation

Finally, a panel of rescue experiments were conducted to illustrate the roles of HADHA/CDK1 in ovarian cancer development. We initiated the investigation by assessing CDK1 levels in both ovarian cancer and normal ovarian epithelial cells, revealing an elevated expression of CDK1 in ovarian cancer cells (Figure S3A). Subsequently, we successfully constructed lentiviruses as indicated, resulting in the downregulation of HADHA and/or the upregulation of CDK1 (Figure S3B and S3C). To gauge the impacts of CDK1 overexpression on cell behavior, we employed a Celigo cell counting assay. The results demonstrated that the overexpression of CDK1 significantly promoted the proliferation of HO8910 and SK-OV-3 cells. Furthermore, it was observed that CDK1 overexpression had the ability to reverse the inhibitory effects of HADHA depletion on cell proliferation (Fig. 4A). Moreover, we examined the influence of CDK1 upregulation on cell migration in HO8910 and SK-OV-3 cells and assessed whether it could mitigate the inhibitory effects of HADHA downregulation. The data presented in Fig. 4B revealed that the upregulation of CDK1 notably enhanced cell migration. Importantly, it also alleviated the inhibitory impact of HADHA downregulation on cell migration.

**Fig. 5:**
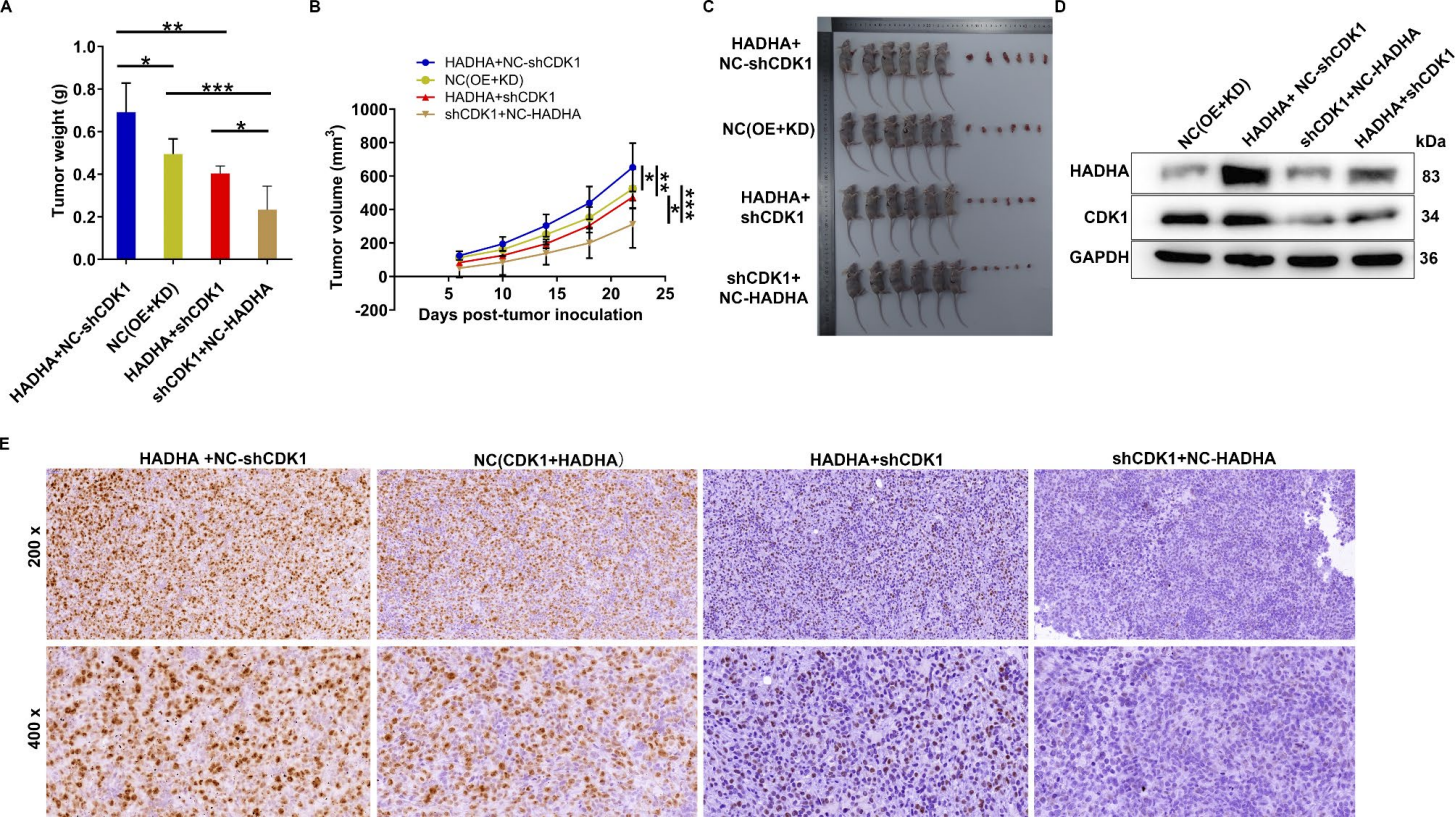
HADHA promoted ovarian cancer tumor outgrowth by upregulating CDK1. **(A, B)** The weight **(A)** and volume **(B)** of tumor, derived from HO-8910 cells with lentiviruses for CDK1 knockdown and/or HADHA overexpression, were measured. **(C)** The photos of tumor collected from mice. **(D)** Western blot assay presented HADHA and CDK1 levels in tumor tissues. **(E)** IHC staining visualized the expression pattern of Ki67 in tumor tissues with CDK1 knockdown and/or HADHA overexpression. NC (OE + KD): Control; CDK1 + NC (KD): CDK1 overexpression; shHADHA + NC (OE): HADHA downregulation; CDK1 + shHADHA: CDK1 overexpression and HADHA downregulation

To determine whether the observed in vitro effects translate to restrained tumor growth in vivo, we conducted subcutaneous implantation of HO-8910 cells treated with the indicated lentiviruses into immune-deficient mice, closely monitoring tumor development. Notably, the primary tumors originating from HADHA-overexpressed cells exhibited a slight increase in size compared to those derived from control cells. Conversely, CDK1-deficient cells resulted in slightly smaller tumors than the control cells, consistent with our in vitro proliferation assays. Most importantly, the inhibition of CDK1 via knockdown partially counteracted the tumor growth enhancement induced by HADHA elevation (Fig. 5A-C). Furthermore, we collected tumor tissues for subsequent western blot assays and immunohistochemical staining. The results unveiled that within tumors derived from HADHA-overexpressed cells, there was a marked abundance of HADHA and Ki67 expression, a marker of cellular proliferation. Intriguingly, this heightened expression of HADHA and Ki67 was partially mitigated by the silencing of CDK1 (Fig. 5D and E). These findings collectively signify that HADHA plays a pivotal role in promoting the progression of ovarian cancer, both in vitro and in vivo, by upregulating CDK1.

## Discussion

In this study, we observed significant overexpression of HADHA in ovarian cancer. Elevated levels of HADHA were correlated with various clinical parameters including pathological grade, tumor size, tumor infiltrate, lymphatic metastasis, distant metastasis, clinical stage and recurrence of state. Besides, HADHA emerged as a prognostic indicator for ovarian cancer patients. Particularly noteworthy was the finding that depleting HADHA resulted in decreased cell proliferation and viability, coupled with an increase in cell apoptosis. Taken together, these findings demonstrated that HADHA functions as a tumor-promoting factor in ovarian cancer.

Then, we delved into the mechanisms through which HADHA influences the progression of ovarian cancer. Our investigations revealed a significant reduction in the levels of CKD1 protein following the downregulation of HADHA. Furthermore, we substantiated the protein interaction between HADHA and CDK1. To shed light on the precise regulatory mechanism by which HADHA and CDK1 are intertwined in mediating ovarian cancer, we probed into the UPS. The UPS plays an important role in cellular processes, particularly in maintaining protein quality control and homeostasis [15]. It is noteworthy that dysregulation of the UPS has been implicated in numerous diseases, including cancer. Indeed, components of the UPS are frequently mutated or abnormally expressed in various cancers [16, 17]. In our study, we made the intriguing discovery that HADHA has the capacity to modulate the ubiquitination of CDK1.

Cyclin-dependent kinases (CDKs) are a group of serine/threonine kinases that collaborate with their corresponding partner cyclins to tightly regulate cell cycle progression. Among all the CDKs, CDK1 holds a unique position as it is essential for key transitions in the cell cycle, including G2/M and G1/S phases, as well as G1 progression [18–20]. To illustrate this point, it’s worth noting that in mouse embryos, the absence of CDK2, CDK3, CDK4, and CDK6 does not impede organogenesis, but the absence of CDK1 leads to failure in this critical developmental process. An expanding body of literature has provided compelling evidence linking elevated levels of CDK1 to the prognosis of various malignant tumors, encompassing lung cancer [21], breast cancer [22], pancreatic cancer [23], cervical cancer [24] and colorectal cancer [25], ovarian cancer [26]. In a relevant study, the depletion of UBE2C was found to reduce the malignancy of ovarian cancer and reverse resistance to cisplatin by downregulating CDK1. In our investigation, we observed that the increased expression of CDK1 had a profound impact on promoting the proliferation and migration of HO8910 and SK-OV-3 cells. Additionally, it counteracted the inhibitory effects of HADHA downregulation on these cellular behaviors. Furthermore, when we knocked down CDK1, we observed a partial restriction in the tumor growth that had been enhanced by elevating HADHA expression. Consequently, we put forth the hypothesis that HADHA facilitates the progression of ovarian cancer by upregulating CDK1, both in vitro and in vivo. This research provides valuable insights into the role of HADHA in ovarian cancer and its potential as a prognostic marker. However, it is essential to acknowledge certain limitations in the study: it requires further clinical validation for HADHA’s prognostic marker potential, needs deeper exploration of molecular mechanisms and signaling pathways involved in HADHA-mediated effects on ovarian cancer progression.

## Conclusions

In summary, our current work demonstrated that unequivocally establishes that HADHA plays a pivotal role in promoting tumorigenesis in ovarian cancer through its modulation of CDK1. This finding underscores the potential significance of HADHA as a promising candidate for targeted therapeutic interventions in the context of ovarian cancer treatment.

### Electronic supplementary material

Below is the link to the electronic supplementary material.

Supplementary Material 1

## Data Availability

The data used and analyzed during the current study are available from the corresponding author on reasonable request.
