# Supplementary material for: HADHA promotes ovarian cancer outgrowth via up-regulating CDK1
Source: Cancer Cell Int. 2023 Nov 20;23:283. doi: 10.1186/s12935-023-03120-4 (PMC10658966; doi:10.1186/s12935-023-03120-4)
Supplement: Supplementary file 1 — Supplementary Material 1 [file 12935_2023_3120_MOESM1_ESM.docx]

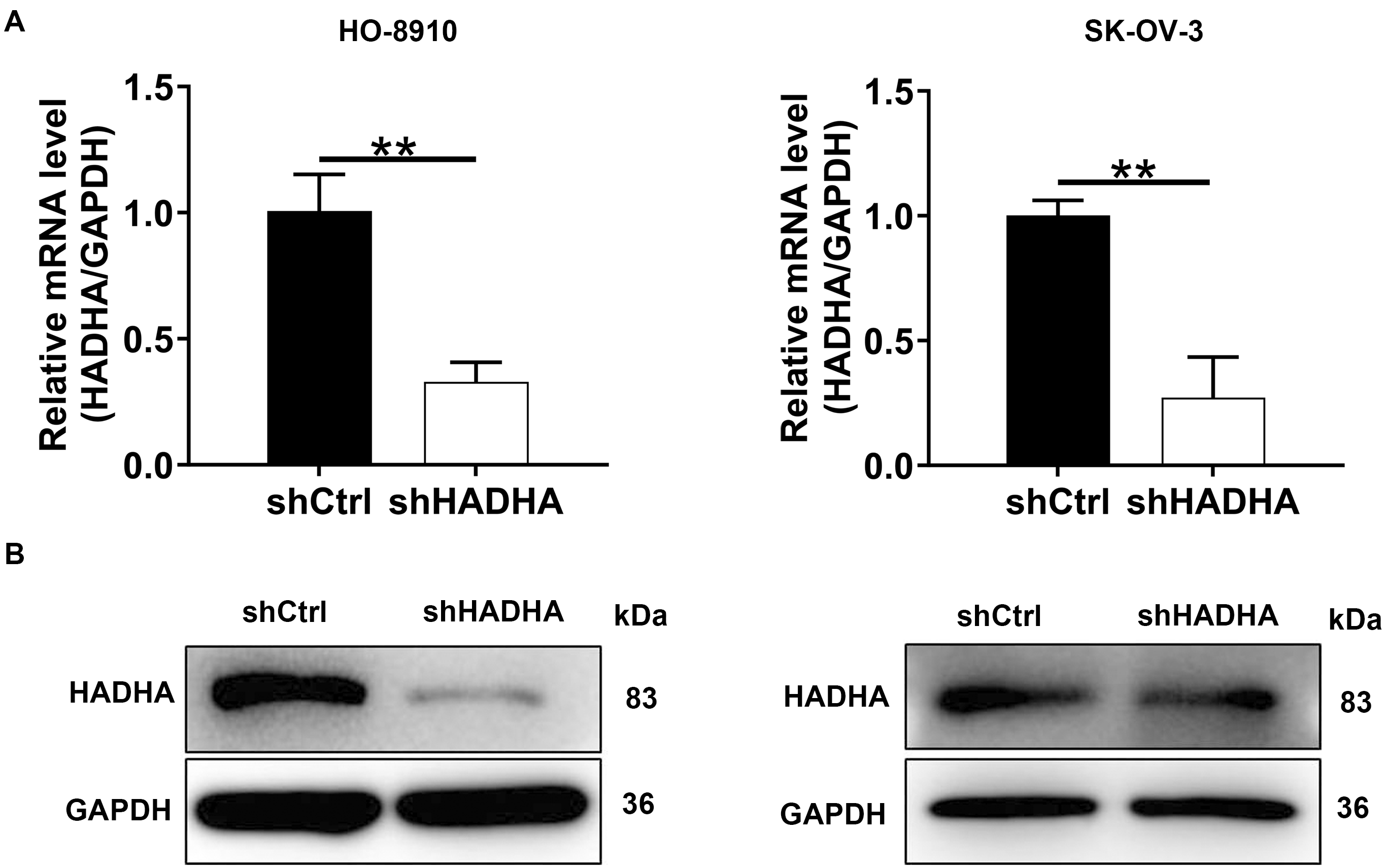


**Figure S1.** (A, B) The mRNA (A) and protein (B) levels of HADHA were detected in HO-8910 and SK-OV-3 cells after knocking down HADHA.


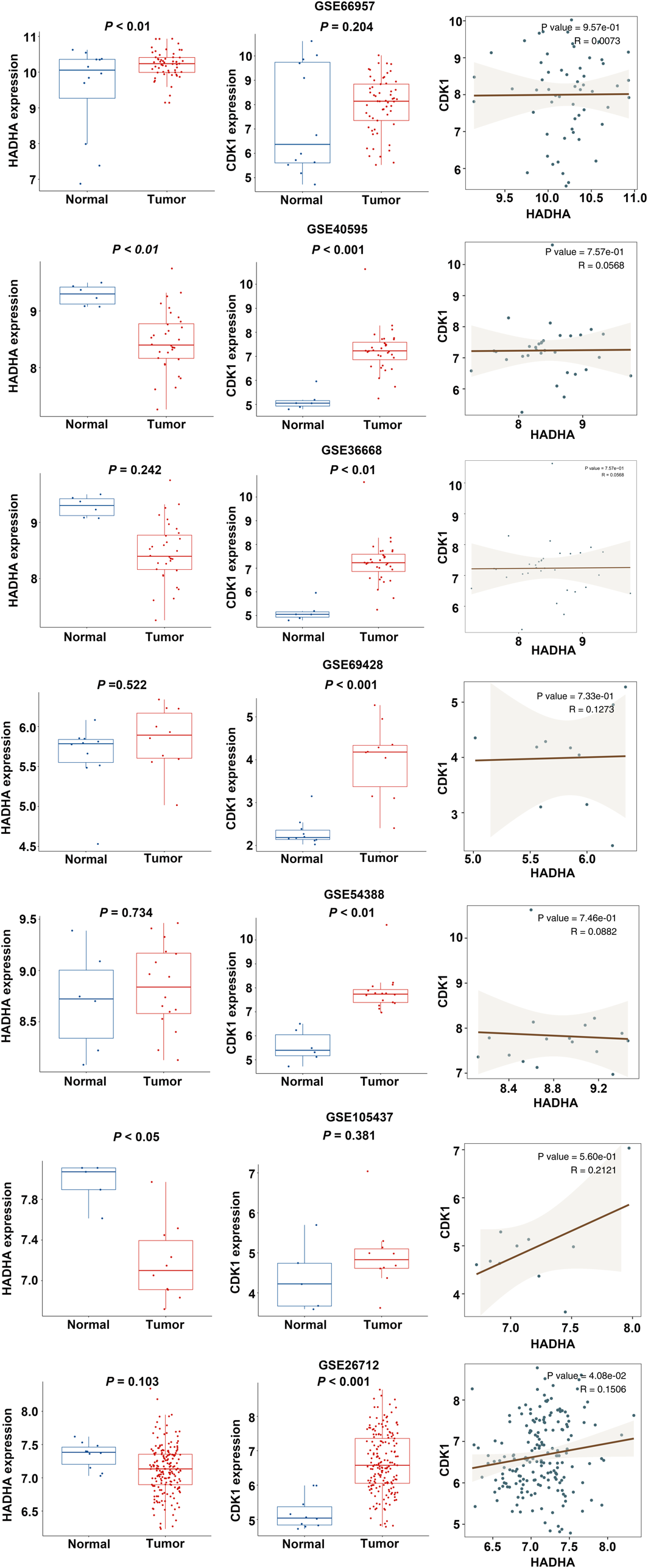


Figure S2. Differential expression analyses of HADHA and CDK1 in ovarian cancer *vs.* normal samples, as well as the examination of expression correlations between HADHA and CDK1, were conducted across multiple datasets retrieved from the Gene Expression Omnibus (GEO) database.


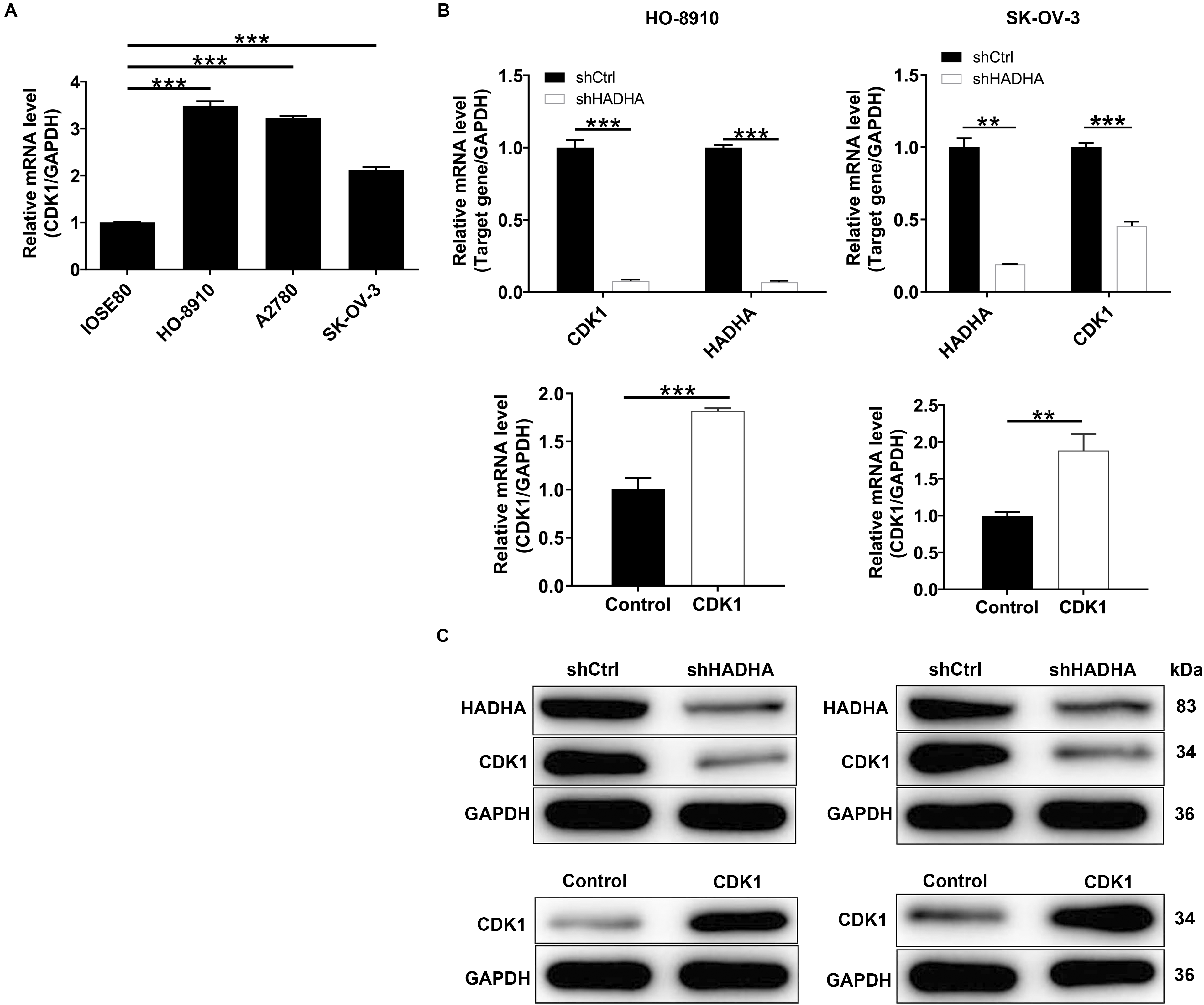


Figure S3. (A) CDK1 mRNA level in three ovarian cancer cell lines HO-8910, A2780, SK-OV-3 cells, as well as normal ovarian epithelial cells IOSE80. (B, C) The mRNA (B) and protein (C) levels of HADHA and CDK1 were detected in HO-8910 and SK-OV-3 cells with lentiviruses for CDK1 knockdown or HADHA overexpression.

Table S1. The detail of antibodies in IHC staining and western blot.

| Primary antibodies | Dilution in IHC | Source species | Company | Catalog No. |
| --- | --- | --- | --- | --- |
| HADHA  Ki67 | 1:300  1:100 | Rabbit  Rabbit | abcam  abcam | ab203114  ab16667 |
| Secondary antibody | Dilution |  | Company | Catalog No. |
| Goat Anti-Rabbit | 1:3000 |  | Beyotime | A0208 |
| Goat Anti- Mouse | 1:3000 |  | Beyotime | A0216 |

| Primary antibodies | Dilution in WB | Source species | Company | Catalog No. |
| --- | --- | --- | --- | --- |
| HADHA | 1:3000 | Rabbit | Proteintech | 10758-1-AP |
| CDK1 | 1:3000 | Rabbit | Abcam | ab265590 |
| PCNA | 1:3000 | Rabbit | Proteintech | 10205-2-AP |
| CAT | 1:1000 | Rabbit | CST | 14097 |
| RAN | 1:1000 | Rabbit | Proteintech | 10469-1-AP |
| PGD | 1:2000 | Rabbit | Proteintech | 14718-1-AP |
| Ubiquitin | 1:1000 | Mouse | Santa Cruz | sc-47721 |
| Secondary antibody | Dilution |  | Company | Catalog No. |
| Goat Anti-Rabbit | 1:3000 |  | Beyotime | A0208 |
| Goat Anti- Mouse | 1:3000 |  | Beyotime | A0216 |

Table S2. The primers in RT-qPCR experiment.

| **Gene** | **Forward primer sequence (5′-3′)** | **Reverse primer sequence (5′-3′)** |
| --- | --- | --- |
| HADHA | CTGCTGGAGATTATCACGACCG | TGATGACCTTCCCCTGCTTG |
| CDK1 | CCATACCCATTGACTAACTAT | ACCCCTTCCTCTTCACTTTC |
| GAPDH | TGACTTCAACAGCGACACCCA | CACCCTGTTGCTGTAGCCAAA |
